# Supplementary material for: Study protocol for a prospective cohort study to investigate Hemodynamic Adaptation to Pregnancy and Placenta-related Outcome: the HAPPO study
Source: BMJ Open. 2019 Nov 10;9(11):e033083. doi: 10.1136/bmjopen-2019-033083 (PMC6858161; doi:10.1136/bmjopen-2019-033083)
Supplement: Author's manuscript [file bmjopen-2019-033083.draft_revisions.pdf]

BMJ Open is committed to open peer review. As part of this commitment we make the peer review history of every article we publish publicly available.

When an article is published we post the peer reviewers' comments and the authors' responses online. We also post the versions of the paper that were used during peer review. These are the versions that the peer review comments apply to.

The versions of the paper that follow are the versions that were submitted during the peer review process. They are not the versions of record or the final published versions. They should not be cited or distributed as the published version of this manuscript.

BMJ Open is an open access journal and the full, final, typeset and author-corrected version of record of the manuscript is available on our site with no access controls, subscription charges or pay-per-view fees (http://bmjopen.bmj.com).

If you have any questions on BMJ Open's open peer review process please email info.bmjopen@bmj.com

BMJ Open

A study protocol for a prospective cohort study to investigate Hemodynamic Adaptation to Pregnancy and Placenta-related Outcome: the HAPPO study

Journal:	*BMJ Open*
Manuscript ID	bmjopen-2019-033083
Article Type:	Protocol
Date Submitted by the Author:	19-Jul-2019
Complete List of Authors:	Bijl, Rianne; Erasmus MC, Obstetrics and Gynaecology<br>Cornette, Jérôme ; Erasmus MC, Obstetrics and Gynaecology<br>van den Bosch, Annemien; Erasmus MC, Cardiology<br>Duvekot, Johannes; Erasmus MC, Obstetrics and Gynaecology<br>Molinger, Jeroen; Erasmus MC, Intensive Care Adults; Duke Medicine, Anesthesiology & Intensive Care medicine, Human Physiology and Pharmacology Lab (HPPL)<br>Willemsen, Sten; Erasmus MC, Obstetrics and Gynaecology; Erasmus MC, Biostatistics<br>Koning, Anton; Erasmus MC, Pathology, Bioinformatics<br>Roos-Hesselink, Jolien; Erasmus MC, Cardiology<br>Steegers-Theunissen, Régine; Erasmus MC, Obstetrics and Gynaecology<br>Franx, Arie; Erasmus MC, Obstetrics and Gynaecology<br>Koster, Maria PH; Erasmus MC, Department of Obstetrics and Gynecology
Keywords:	Pregnancy complications, Placental development, Preconception, Cardiovascular health, Adaptation

SCHOLARONE™
Manuscripts

A study protocol for a prospective cohort study to investigate Hemodynamic Adaptation to Pregnancy and Placenta-related Outcome: the HAPPO study

R.C. Bijl[1], J.M.J. Cornette[1], A.E. van den Bosch[2], J.J. Duvekot[1], J. Molinger[3,4,5], S.P. Willemsen[1,6], A.H.J. Koning[7], J.W. Roos-Hesselink[2], A. Franx[1], R.P.M. Steegers-Theunissen[1], M.P.H. Koster[1]

[1] Department of Obstetrics and Gynaecology, Erasmus MC, Rotterdam, the Netherlands

[2] Department of Cardiology, Erasmus MC, Rotterdam, the Netherlands

[3] Department of Intensive Care Adults, Erasmus MC, Rotterdam, the Netherlands

[4] BeLife Clinical Human Performance Centre, Rotterdam, the Netherlands

[5] Department of Anaesthesiology & Intensive Care medicine, Human Physiology and Pharmacology Lab (HPPL). Duke University Medical Centre, Durham, NC, United States

[6] Department of Biostatistics, Erasmus MC, Rotterdam, the Netherlands

[7] Department of Pathology, Bioinformatics, Erasmus MC, Rotterdam, the Netherlands

Correspondence to: dr. M.P.H. Koster, m.p.h.koster@erasmusmc.nl, +31 10 704 0 704

Erasmus MC, University Medical Centre, PO Box 2040, 3000 CA Rotterdam, the Netherlands

Word count: 3399

For peer review only

ABSTRACT

Introduction

The importance of cardiovascular health in relation to pregnancy outcome is increasingly acknowledged. Women who develop certain pregnancy complications, in particular preeclampsia, are at higher risk for future cardiovascular disease. Independent of its outcome, pregnancy requires a substantial adaptive response of the maternal cardiovascular system. In the HAPPO study, we aim to examine longitudinal maternal hemodynamic adaptation to pregnancy from the preconception period onwards. We hypothesize that women who will develop adverse pregnancy outcomes have impaired cardiovascular health before conception, leading to hemodynamic maladaptation to pregnancy and diminished uteroplacental vascular development.

Methods and analysis

In this prospective cohort study embedded in the Rotterdam periconception cohort, 200 women with a history of placenta-related pregnancy complications (high-risk group) and 100 women with an uncomplicated obstetric history (low-risk group) will be included. At five moments (preconception, first-, second- and third trimester, and post-delivery), women will undergo an extensive examination of the macro- and microcirculatory system and uteroplacental vascular development. The main outcome measures are differences in maternal hemodynamic adaptation to pregnancy between women with and without placenta-related pregnancy complications. In a multivariate linear mixed model, the relationship between maternal hemodynamic adaptive parameters, (utero)placental vascularization indices and clinical outcomes (occurrence of pregnancy complications, embryonic and foetal growth trajectories, miscarriage rate, gestational age at delivery, birth weight) will be studied. Subgroup analysis will be performed to study baseline and trajectory differences between high- and low-risk women, independent of subsequent pregnancy outcome.

Ethics and dissemination

This study protocol was approved by the Medical Ethics Committee of the Erasmus MC , Rotterdam, the Netherlands (MEC 2018-150). Results will be disseminated to the medical community by publications in peer-reviewed journals and presentations at scientific

For peer review only

congresses. Also, patient associations will be informed and the public will be informed by dissemination through (social) media.

Registration details

Trial NL7394

Keywords

Pregnancy complications, placental development, preconception, cardiovascular health, adaptation

For peer review only

ARTICLE SUMMARY

Strengths and limitations of this study

- An integrated approach consisting of parallel non-invasive techniques assessing multiple hemodynamic parameters will compile extensive hemodynamic profiles of participants.
- Hemodynamic adaptation to pregnancy will be measured prospectively from the preconception period onwards to three months post-delivery, thereby covering an important window in the reproductive timespan.
- Simultaneous assessment of maternal hemodynamic adaptation to pregnancy and uteroplacental vascular development will provide new insights into the interaction between both mechanisms and the pathophysiology of pregnancy complications.
- Since only multiparous women will be included, conclusions about whether maternal hemodynamic maladaptation is a cause or consequence of previous pregnancy outcome cannot be provided.

For peer review only

INTRODUCTION

Context

Placenta-related pregnancy complications (PPC), such as preeclampsia and/or foetal growth restriction, occur in approximately 10-15% of all pregnancies.[1-3] PPC not only affect perinatal morbidity and mortality but also significantly impact the long-term cardiovascular health of women and their children with associated health care costs for society.[4-6] For example, women with a history of preeclampsia have a two- to sevenfold increased risk to develop ischemic heart disease in later life, depending on the gestational age and severity at onset of preeclampsia.[4] Thus, early identification of women who are at risk for PPC is not only important to enable subsequent personalized prevention and treatment strategies in order to improve reproductive outcome, but also to improve lifelong cardiovascular health. To date, most available screening tests for adverse pregnancy outcome use blood pressure and uterine artery blood flow, which reflect the hemodynamic profile of the mother, and thereby the likelihood of impaired placental development and subsequent adverse events. Nonetheless, these markers are not directly related to cardiac or placental function, which may explain the moderate predictive performances of most prognostic models.[7 8]

Hemodynamic adaptation to pregnancy

Pregnancy requires a physiological adaptive response of the maternal cardiovascular system with a substantial rise in plasma volume and cardiac output (CO).[9] Already in the first trimester of pregnancy, a woman's systemic vascular resistance decreases with 35-40% and her stroke volume increases with 8%.[9] This natural volume overload leads to a reversible, physiological left ventricular dilatation and a possible change in left ventricular diastolic function.[10] In most pregnancies, these changes occur without clinical problems. However, in women who develop PPC, maternal hemodynamic adaptation can be different and diastolic dysfunction is more often present.[11 12]

Moreover, in women with complicated pregnancies, endothelial dysfunction and increased vascular stiffness are also more common.[13 14] The endothelial lining of the vasculature in the human body is of great importance in the regulation of vascular tone. Vascular stiffness increases during normal aging by the replacement of elastin fibres with less elastic collagen. Endothelial function tests and arterial vascular stiffness measurements have already proven to be of importance in predicting cardiovascular disease.[15]

For peer review only

Together, this all supports the hypothesis that in women who develop PPC an increased risk for cardiovascular disease is unmasked by the stressed state of pregnancy.[16 17] However, knowledge on maternal hemodynamic adaptation to pregnancy, as early as the time of conception, is scarce and often only focuses on one or two parameters instead of the complex interaction between macro- and microcirculatory parameters. Only a few studies starting prior to pregnancy have shown hemodynamic changes in, for example, blood pressure, heart rate and CO early in pregnancy.[18-22] An integrated approach using parallel techniques and multiple hemodynamic parameters from the preconception period onwards throughout pregnancy is needed to provide insight in individual (patho)physiological hemodynamic adaptation to pregnancy.

Placental development and function

The adequate establishment of the maternal–foetal vascular interface early in gestation is essential for a healthy pregnancy outcome. Among others, this requires adaptation of the uterine spiral arteries into remodelled vessels to create a low-resistance uteroplacental circulation.[23 24] Failure in the adaptation of the uterine vasculature causes hypoperfusion of the placenta and is widely believed to underlie several pregnancy complications.[25 26] This is substantiated by several changes in histopathology, including decidual vasculopathy, villous hypoplasia and lesions consistent with loss of integrity of the vascular wall of the maternal circulation in delivered placentas of women with PPC.[27-29] However, the aetiology of these placental abnormalities and its relation with maternal cardiovascular health remains unclear.

RESEARCH HYPOTHESIS AND AIM

We hypothesize that impaired maternal cardiovascular health leads to hemodynamic maladaptation to pregnancy, diminished uteroplacental vascular development and subsequent pregnancy complications (Figure 1). The overall aim of the HAPPO study is to examine associations between maternal hemodynamic adaptation to pregnancy and (utero)placental vascular development in women with and without pregnancy complications. Therefore, we specified the following objectives:

1. To assess hemodynamic parameters before, during and after pregnancy to gain insight in (patho)physiological maternal hemodynamic adaptation mechanisms;

For peer review only

2. To assess longitudinal utero(placental) vasculature development using state-of-the-art three-dimensional ultrasound and Virtual Reality techniques combined with *in vivo* examination of placental bed biopsies;
3. To construct a multivariate model to study the associations between maternal hemodynamic adaptation to pregnancy, utero(placental) vascular development and PPC.
4. To study differences in baseline hemodynamic parameters and hemodynamic adaptation to pregnancy between women with and without a history of PPC .

STUDY DESIGN

The HAPPO study is a single-centre prospective cohort study, embedded in the ongoing Rotterdam periconception cohort, performed at the department of Obstetrics and Gynaecology of the Erasmus MC, University Medical Centre Rotterdam, the Netherlands.[30]

STUDY POPULATION

We aim to include 200 women with a history of pregnancy complications (i.e. preeclampsia and/or children born small for gestational age [SGA]). Preeclampsia is defined according to the American College of Obstetricians and Gynecologists (ACOG) criteria and SGA is defined as birthweight below the 10th percentile according to the Dutch growth charts (based on the Hoftiezer charts).[1 2 31 32] Women are also eligible for inclusion when their neonate was born after induction of labour for suspected foetal growth restriction and foetal Doppler abnormalities. Doppler abnormalities are defined as umbilical artery pulsatility index >95th percentile, absent or reversed end-diastolic flow in the umbilical artery or a cerebroplacental ratio <1.[33]

Also, 100 women after uncomplicated pregnancies and (term) deliveries will be included as a low-risk control group. Women are eligible to participate in this study when they have a minimum age of 18 years, the delivery date of their last pregnancy was more than one year ago, they have a current wish to become pregnant and the previous pregnancy was either complicated by PPC *or* the previous pregnancy was uncomplicated. Women who are unable or unwilling to provide informed consent will be excluded from participation. Also, if a woman

For peer review only

is currently breastfeeding, she will be excluded from participation since this may influence her vascular condition. The in- and exclusion criteria are shown in Table 1.

Table 1. Inclusion and exclusion criteria for participants in the HAPPO study.

Inclusion criteria	▪ Minimum age of 18 years
	▪ Delivery date of last pregnancy more than 1 year ago
	▪ Previous pregnancy complicated by PPC *or* uncomplicated previous pregnancy
	▪ Current wish to become pregnant
Exclusion criteria	▪ Unable or unwilling to provide informed consent
	▪ Currently breastfeeding

STUDY PROCEDURES/MEASUREMENTS

An overview of all study procedures is provided in Figure 2. The first study visit will be planned when the participating woman has the wish to become pregnant, but before conception takes place. When a pregnancy occurs within one year, a study visit will be planned in each trimester as well as three months post-delivery, which will be the last study visit.

Questionnaires

Participants of the study will receive a total of three questionnaires: 1) after the preconceptional study visit, participants will receive an online questionnaire on demographic characteristics, medical conditions and lifestyle; 2) preceding the study visit in the first trimester of pregnancy, participants will receive an online questionnaire to address possible changes in medical conditions and lifestyle; 3) after delivery, participants will receive an online follow-up questionnaire on pregnancy and birth outcomes (i.e. gestational age at birth, mode of delivery, birth weight, neonatal sex, pregnancy and/or delivery complications).

Anthropometrics

At each visit, a woman’s height, weight, hip and waist circumference will be measured using standardized, validated methods to compose the classical cardiovascular risk factors body mass index (BMI) and waist-to-hip ratio.[34]

Blood sample

For peer review only

Two blood samples (8.5mL serum tube and 10mL EDTA tube) will be obtained at the preconception visit, the first- and third-trimester visit, and the post-delivery visit. All samples will be centrifuged and divided into aliquots of serum, plasma, whole blood and buffy coat and then stored in a -80°C freezer at the trial laboratory at our own facility for future assessment of cardiovascular markers (e.g. CRP and lipids) and markers of placental function (e.g. PlGF and sFlt-1).

Echocardiography

Transthoracic echocardiography has become the preferential method for safe, non-invasive cardiac assessment in pregnant women.[35 36] At all study visits, echocardiography will be performed using an Affiniti 70G ultrasound machine (Philips Healthcare, Andover, United States of America) by a trained researcher (R.C.B.) under the supervision of a cardiologist (A.E.B.). Echocardiograms will be fulfilled according to the recommendations of the European Association of Cardiovascular imaging.[37] CO will be derived from the left ventricle outflow tract velocity time integral (LVOT VTI) and LVOT cross-sectional area (LVOT CSA). From this, systemic vascular resistance will be calculated (i.e. mean blood pressure divided by CO). Left ventricular diastolic function will be assessed by mitral inflow (E/A) and tissue Doppler (E/e') examination. Global left ventricular systolic function will be assessed by using the Teichholz method for ejection fraction calculation.[38]

Post occlusion reactive hyperaemia

One of the first manifestations of an impaired cardiovascular risk profile is endothelial dysfunction of small vessels (microcirculation). Post occlusion reactive hyperaemia (PORH) will be used in this study to assess the endothelial function of the peripheral microvasculature. We use devices from Moor instruments (moorVMS-VASC 2.0, moorVMS-PRES and moorVMS-LDF; Axminster, United Kingdom) to measure microvascular blood flow reactivity following arterial occlusion by using a non-invasive laser Doppler flowmetry (LDF) technique. In resting position, after three minutes of arterial occlusion by a cuff surrounding the upper left arm, the reperfusion of microvascular blood in the skin is measured by the LDF skin probe which is attached to the lower arm. The peak response after the release of the occlusion and the time to recover to baseline reflect endothelial regulated microvascular function.[39 40]

For peer review only

Pulse wave analysis

A cuff-based oscillometric device (Mobil-O-Graph® PWA by I.E.M., Stolberg, Germany) will be used to assess central and peripheral blood pressure, pulse wave velocity and augmentation index (AIx).[41 42] Pulse wave velocity values are derived from an algorithm which integrates age, central systolic blood pressure and data derived from pulse wave analysis into a mathematical model.[43] Higher values of pulse wave velocity indicate higher arterial stiffness and thus a worse vascular condition. The AIx provides additional information on the stiffness of the aorta and is derived from the central pressure waveform.

Cardiopulmonary exercise test with bio-impedance cardiography

To assess the hemodynamic response during physical stress, CO will also be measured using signal morphology impedance cardiography (SM-ICG) using PhysioFlow® PF07 Enduro (Manatec biomedical, Poissy, France). The exercise during which the SM-ICG will be performed, is a cardiopulmonary exercise test (CPET) on a cycle ergometer with a RAMP protocol to 70% of the estimated maximum heart rate according to the Tanaka formula.[44] Moderate exercise limited to 70% of the maximal heart rate is considered safe during pregnancy.[45]

Uteroplacental vascularization measurements

Studies using conventional two-dimensional (2D) Doppler ultrasound have shown that increased vascular resistance of the uterine arteries is associated with an increased risk for subsequent development of PPC.[7 46] Therefore, pulsed wave Doppler measurements of the right and left uterine artery will be performed at all study visits to provide the pulsatility and resistance indices. Moreover, to evaluate the morphology of the uterine and placental vasculature and quantification of perfusion, a 3D power Doppler scan of the uterine (preconception visit) and placental bed (first-trimester visit) vasculature will be performed, also using the Affiniti 70G ultrasound machine. By assessing these 3D datasets in an innovative virtual reality (VR) system (Barco I-Space or 3D desktop), depth perception and three dimensional interaction enables more accurate and detailed quantification of the utero(placental) vascular volumes. At the Erasmus MC there is extensive experience with this novel technology and the uteroplacental vascular volume measurements have proven to be feasible and reliable.[47 48]

For peer review only

With respect to the risks of performing ultrasound examinations in the first trimester using 3D power Doppler, recommendations as set by the International Society of Ultrasound in Obstetrics and Gynaecology (ISUOG) are followed for the purpose of this study (i.e. thermal index (TI) ≤ 1.0, mechanical index (MI) <1.9 and total exposure time < 10 min). Transvaginal scanning time (preconception and first trimester) and transabdominal scanning time (second and third trimester) will be kept as short as possible according to the ALARA principles.[49 50]

Embryonic and foetal growth parameters

At the first trimester visit, additional parameters of embryonic growth will be measured, i.e. crown-rump length and embryonic volume. The obtained 3D datasets will be visualized in the Barco I-Space or desktop version and offline volume measurements will be performed.

At the second and third trimester visit, additional parameters of foetal growth (head circumference, abdominal circumference, femur length) and pulsed wave Doppler measurements of the umbilical artery and median cerebral artery will be performed by transabdominal ultrasound.

Placental examinations

At delivery, histological samples of *in vivo* placental vasculature will be harvested. The placenta will be collected according to standard procedures and four samples of approximately 3cm*3cm are then fixated in 4% paraformaldehyde for storage. The fixated placenta biopsies will be processed and studied as done in routine placental pathology examination.

In a subgroup of women who undergo a caesarean section (estimated 30% of our study population) at the Erasmus MC, four punch biopsies of the placental bed will be collected, which consist of decidual and myometrial tissue and contain spiral arteries.[51] The samples are directly rinsed with sterile NaCl to wash excessive blood and then fixated in 4% paraformaldehyde for storage and examination. According to our previously published protocol, several markers will be used to score the developmental state of the spiral arteries, identifying typical acute atherosis lesions and evaluating involvement of the immune system.[51 52]

OUTCOME MEASURES

For peer review only

The primary outcome measure of the HAPPO study is the difference in maternal hemodynamic adaptation to pregnancy, expressed as the trajectory of CO assessed by echocardiography before, during and after pregnancy, between women who do and do not develop PPC. CO is one of the most frequently described hemodynamic parameters in pregnancy, alongside with blood pressure. By using transthoracic echocardiography, as a validated and safe method to measure CO, we will obtain consecutive and reliable information on maternal hemodynamic adaptation to pregnancy.[36]

The secondary outcome measures are differences in additional indices of maternal hemodynamic adaptation to pregnancy and utero(placental) vascular development between women who do and do not develop PPC. This includes differences in weight gain during pregnancy, systemic vascular resistance, left ventricular diastolic function, endothelial regulated microvascular function, pulse wave analysis, hemodynamic response to exercise, placental vascular volume, foetal growth parameters (including Doppler measurements) and placenta/placental bed pathology examinations.

SAMPLE SIZE CALCULATION

Since we aim to investigate multiple hemodynamic parameters and literature on this subject is scarce, sample sizes are difficult to calculate. There are a few studies that describe the longitudinal trajectory of CO from preconception until after delivery in uncomplicated pregnancies and a couple of cross-sectional studies that investigated CO in either the first of third trimester of pregnancy.[18-20 54 55] Based on these studies, we expect a difference of approximately 0.5 standard deviation (SD) at each time point and an intraclass correlation coefficient of at least 0.3 between time points in our study. With alpha set at 0.05 and a power of 0.8, simulation for linear mixed model analysis based on these estimates showed that 38 cases (PPC in the anticipated pregnancy) are needed to establish such differences.[56]

Based on literature, we expect a 25% recurrence rate of PPC in the high-risk group and a 2% incidence of PPC in the low-risk group.[57-59] Thus, PPC cases will primarily occur in the high-risk group. For an expected number of 38 cases we would need to follow-up 38/0.25=152 pregnancies in the high-risk group.

Multiparous women who wish to become pregnant who are actively contemplating pregnancy, are assumed to have an approximately 85% chance to become pregnant again within one year.[60 61] The overall miscarriage rate in this study group is estimated to be

For peer review only

approximately 10%.[62] With this taken into account, we would need to include 152/(0.85*0.90)=198 high-risk women prior to conception. For comparison with women at low risk for PPC and to increase the number of controls, we will also include 100 low-risk women with an uncomplicated previous pregnancy. With this sample size we expect, based on previous research, to be able to detect differences of approximately 5-10% in other hemodynamic parameters and utero(placental) vasculature development (secondary outcomes).[11 14 18 54 63-69]

STATISTICAL ANALYSIS PLAN

Patient characteristics, medical conditions and lifestyle data will be presented as means [SD] or numbers [percentage] and compared using t-tests and chi-square tests. Hemodynamic parameters will be presented as means [SD] at each time point and compared between groups using t-tests.

Multivariate linear mixed models will be used to analyse the trajectories of maternal hemodynamic parameters over time. We will test whether there is a difference between women who do and do not develop PPC by comparing each trajectory using a likelihood ratio test.

Subgroup analyses will be performed to study (baseline and trajectory) differences between high- and low-risk women, independent of subsequent pregnancy outcome. Bonferroni corrections will be applied to control for multiplicity of secondary outcome parameters.

In case of significant differences in patient characteristics between groups, regression analyses will be adjusted for these characteristics.

DATA COLLECTION, HANDLING AND STORAGE

The study will be performed according to written informed consent procedures which ensures personal data protection and confidentiality. Data will be de-identified (pseudo-anonymized) in order to guarantee the privacy of the participants, the key to the code will be safeguarded by the investigators and will remain on site with the local research team.

All research data will be retained and stored in the HAPPO-database at a secured server at the research institution and it will remain there for at least 15 years after completion of the

For peer review only

project. All imaging data will be stored and handled in the same way and according to the same rules and regulations as other patient-related imaging data at Erasmus MC.

Monitoring of data and safety will be performed once a year, according to local protocol for research with negligible risk.

PATIENT AND PUBLIC INVOLVEMENT

Patient and public were not involved in the design of the study. However, at the earliest moment of recruitment, two large patient associations for women who experienced PE and/or HELLP syndrome (i.e. HELLP Stichting [www.hellp.nl] and the Preeclampsia Foundation [www.preeclampsia.org]) were informed and are involved in promoting the HAPPO study.

ETHICS AND DISSEMINATION

The study will be conducted according to the principles of the Declaration of Helsinki (version October 2013) and in accordance with relevant national guidelines, regulations and Acts (e.g. the Medical Research Involving Human Subjects Act [WMO]). The study protocol was approved by the Medical Ethics Committee of the Erasmus MC (MEC-2018-150). The study is registered in the Dutch Trial Registry (NL7394).

The HAPPO study team invites colleagues who are interested in collaboration or data sharing to contact the principal investigators at HAPPO.studie@erasmusmc.nl.

Results of the study will be disseminated to healthcare professionals and to scientific and industrial peers through events, professional organizations and publications in peer-reviewed (preferably open-access) journals as well as presentations at scientific congresses. Also, results will be communicated to women who wish to become pregnant and women who experienced pregnancy complications by informing patient associations and by dissemination via (social) media.

FUTURE IMPLICATIONS

The results of this study will provide more understanding of longitudinal maternal hemodynamic adaptation to pregnancy in relation to utero(placental) vascular development and its associations with subsequent pregnancy outcome. Hereby, starting points for future possibilities to optimize cardiovascular and placental health by interventions in clinical

For peer review only

validation studies will arise. This will enable the development of more accurate, personalized prevention and treatment strategies for women with high-risk pregnancies, from the preconception period onwards. Thereby, we aim to reduce subsequent maternal and perinatal morbidity and mortality in the future, eventually leading to life-long reduced risks and costs of cardiovascular diseases.

AUTHOR'S CONTRIBUTIONS

The rationale and design of the study were conceived by JMJC, JJD, JWRH, RPMST and MPHK. AEB, JM and AHJK contribute their invaluable practical expertise and SPW his excellent statistical expertise. RCB is the executive researcher, supervised by JMJC, AF, and MPHK. MPHK is the principle investigator of the study and responsible for all aspects of the study. RCB and MPHK prepared the first draft of the protocol. All authors have read and approved the final manuscript.

FUNDING STATEMENT

This work is supported by an Erasmus MC Fellowship Grant (2017) and the Peter Joseph Pappas Research Grant from the Preeclampsia Foundation (2019), both awarded to dr. M.P.H. Koster. These sponsors had no involvement in the design of the study.

COMPETING INTEREST STATEMENT

None declared.

For peer review only

FULL REFERENCES

1. ACOG Practice Bulletin No. 202: Gestational Hypertension and Preeclampsia. Obstet Gynecol 2019;**133**(1):e1-e25.
2. ACOG Practice Bulletin No. 204: Fetal Growth Restriction. Obstet Gynecol 2019;**133**(2):e97-e109.
3. Steegers EA, von Dadelszen P, Duvekot JJ, Pijnenborg R. Pre-eclampsia. Lancet 2010;**376**(9741):631-44.
4. Bellamy L, Casas JP, Hingorani AD, Williams DJ. Pre-eclampsia and risk of cardiovascular disease and cancer in later life: systematic review and meta-analysis. Bmj 2007;**335**(7627):974.
5. Veerbeek JH, Hermes W, Breimer AY, van Rijn BB, Koenen SV, Mol BW, Franx A, de Groot CJ, Koster MP. Cardiovascular disease risk factors after early-onset preeclampsia, late-onset preeclampsia, and pregnancy-induced hypertension. Hypertension 2015;**65**(3):600-6.
6. Grandi SM, Filion KB, Yoon S, Ayele HT, Doyle CM, Hutcheon JA, Smith GN, Gore GC, Ray JG, Nerenberg K, Platt RW. Cardiovascular Disease-Related Morbidity and Mortality in Women With a History of Pregnancy Complications. Circulation 2019;**139**(8):1069-79.
7. Townsend R, Khalil A, Premakumar Y, Allotey J, Snell KIE, Chan C, Chappell LC, Hooper R, Green M, Mol BW, Thilaganathan B, Thangaratinam S, Network I. Prediction of pre-eclampsia: review of reviews. Ultrasound Obstet Gynecol 2018.
8. Lamain-de Ruiter M, Kwee A, Naaktgeboren CA, Louhanepessy RD, De Groot I, Evers IM, Groenendaal F, Hering YR, Huisjes AJM, Kirpestein C, Monincx WM, Schielen P, Van 't Zelfde A, Van Oirschot CM, Vankan-Buitelaar SA, Vonk M, Wiegers TA, Zwart JJ, Moons KGM, Franx A, Koster MPH. External validation of prognostic models for preeclampsia in a Dutch multicenter prospective cohort. Hypertens Pregnancy 2019;**38**(2):78-88.
9. Cornette J, Roos-Hesselink JW. Normal Cardiovascular Adaptation to Pregnancy. In: Stergiopoulos K, Brown DL, eds. Evidence-Based Cardiology Consult. London: Springer London, 2014:423-32.
10. Schannwell CM, Zimmermann T, Schneppenheim M, Plehn G, Marx R, Strauer BE. Left ventricular hypertrophy and diastolic dysfunction in healthy pregnant women. Cardiology 2002;**97**(2):73-8.
11. Bosio PM, McKenna PJ, Conroy R, O'Herlihy C. Maternal central hemodynamics in hypertensive disorders of pregnancy. Obstet Gynecol 1999;**94**(6):978-84.
12. Bamfo JE, Kametas NA, Turan O, Khaw A, Nicolaides KH. Maternal cardiac function in fetal growth restriction. Bjog 2006;**113**(7):784-91.
13. Mannaerts D, Faes E, Goovaerts I, Stoop T, Cornette J, Gyselaers W, Spaanderman M, Van Craenenbroeck EM, Jacquemyn Y. Flow-mediated dilation and peripheral arterial tonometry are disturbed in preeclampsia and reflect different aspects of endothelial function. Am J Physiol Regul Integr Comp Physiol 2017;**313**(5):R518-R25.
14. Osman MW, Nath M, Breslin E, Khalil A, Webb DR, Robinson TG, Mousa HA. Association between arterial stiffness and wave reflection with subsequent development of placental-mediated diseases during pregnancy: findings of a systematic review and meta-analysis. J Hypertens 2018;**36**(5):1005-14.
15. Eastabrook G, Aksoy T, Bedell S, Penava D, de Vrijer B. Preeclampsia biomarkers: An assessment of maternal cardiometabolic health. Pregnancy Hypertens 2018;**13**:204-13.
16. Sattar N, Greer IA. Pregnancy complications and maternal cardiovascular risk: opportunities for intervention and screening? Bmj 2002;**325**(7356):157-60.
17. Thilaganathan B, Kalafat E. Cardiovascular System in Preeclampsia and Beyond. Hypertension 2019;**73**(3):522-31.
18. Meah VL, Cockcroft JR, Backx K, Shave R, Stohr EJ. Cardiac output and related haemodynamics during pregnancy: a series of meta-analyses. Heart 2016;**102**(7):518-26.
19. Mahendru AA, Everett TR, Wilkinson IB, Lees CC, McEniery CM. Maternal cardiovascular changes from pre-pregnancy to very early pregnancy. J Hypertens 2012;**30**(11):2168-72.

For peer review only

20. Mahendru AA, Foo FL, McEniery CM, Everett TR, Wilkinson IB, Lees CC. Change in maternal cardiac output from preconception to mid-pregnancy is associated with birth weight in healthy pregnancies. Ultrasound Obstet Gynecol 2017;**49**(1):78-84.
21. Foo FL, Mahendru AA, Masini G, Fraser A, Cacciatore S, MacIntyre DA, McEniery CM, Wilkinson IB, Bennett PR, Lees CC. Association Between Prepregnancy Cardiovascular Function and Subsequent Preeclampsia or Fetal Growth Restriction. Hypertension 2018;**72**(2):442-50.
22. Ghossein-Doha C, Spaanderman ME, Al Doulah R, Van Kuijk SM, Peeters LL. Maternal cardiac adaptation to subsequent pregnancy in formerly pre-eclamptic women according to recurrence of pre-eclampsia. Ultrasound Obstet Gynecol 2016;**47**(1):96-103.
23. Regnault TR, Galan HL, Parker TA, Anthony RV. Placental development in normal and compromised pregnancies-- a review. Placenta 2002;**23 Suppl A**:S119-29.
24. Burton GJ, Jauniaux E. Pathophysiology of placental-derived fetal growth restriction. Am J Obstet Gynecol 2018;**218**(2S):S745-S61.
25. Burton GJ, Charnock-Jones DS, Jauniaux E. Regulation of vascular growth and function in the human placenta. Reproduction 2009;**138**(6):895-902.
26. Fisher SJ. Why is placentation abnormal in preeclampsia? Am J Obstet Gynecol 2015;**213**(4 Suppl):S115-22.
27. Lockwood CJ, Krikun G, Caze R, Rahman M, Buchwalder LF, Schatz F. Decidual cell-expressed tissue factor in human pregnancy and its involvement in hemostasis and preeclampsia-related angiogenesis. Ann N Y Acad Sci 2008;**1127**:67-72.
28. Predoi CG, Grigoriu C, Vladescu R, Mihart AE. Placental damages in preeclampsia - from ultrasound images to histopathological findings. J Med Life 2015;**8 Spec Issue**:62-5.
29. Travaglino A, Raffone A, Saccone G, Migliorini S, Maruotti GM, Esposito G, Mollo A, Martinelli P, Zullo F, D'Armiento M. Placental morphology, apoptosis, angiogenesis and epithelial mechanisms in early-onset preeclampsia. Eur J Obstet Gynecol Reprod Biol 2019;**234**:200-06.
30. Steegers-Theunissen RP, Verheijden-Paulissen JJ, van Uitert EM, Wildhagen MF, Exalto N, Koning AH, Eggink AJ, Duvekot JJ, Laven JS, Tibboel D, Reiss I, Steegers EA. Cohort Profile: The Rotterdam Periconceptional Cohort (Predict Study). Int J Epidemiol 2016;**45**(2):374-81.
31. Perined. Geboortegewichtcurven: Perined; [Available from: https://www.perined.nl/producten/geboortegewichtcurven.
32. Hoftiezer L, Hof MHP, Dijs-Elsinga J, Hogeveen M, Hukkelhoven CWPM, van Lingen RA. From population reference to national standard: new and improved birthweight charts. American Journal of Obstetrics & Gynecology 2019;**220**(4):383.e1-83.e17.
33. Figueras F, Caradeux J, Crispi F, Eixarch E, Peguero A, Gratacos E. Diagnosis and surveillance of late-onset fetal growth restriction. Am J Obstet Gynecol 2018;**218**(2S):S790-S802 e1.
34. de Koning L, Merchant AT, Pogue J, Anand SS. Waist circumference and waist-to-hip ratio as predictors of cardiovascular events: meta-regression analysis of prospective studies. Eur Heart J 2007;**28**(7):850-6.
35. Cornette J, Laker S, Jeffery B, Lombaard H, Alberts A, Rizopoulos D, Roos-Hesselink JW, Pattinson RC. Validation of maternal cardiac output assessed by transthoracic echocardiography against pulmonary artery catheterization in severely ill pregnant women: prospective comparative study and systematic review. Ultrasound Obstet Gynecol 2017;**49**(1):25-31.
36. Bijl RC, Valensise H, Novelli GP, Vasapollo B, Wilkinson I, Thilaganathan B, Stöhr EJ, Lees C, van der Marel CD, Cornette JMJ, on behalf of the International Working Group on Maternal H. Methods and considerations concerning cardiac output measurement in pregnant women: recommendations of the International Working Group on Maternal Hemodynamics. Ultrasound in Obstetrics & Gynecology 2019;**54**(1):35-50.
37. Nagueh SF, Smiseth OA, Appleton CP, Byrd BF, 3rd, Dokainish H, Edvardsen T, Flachskampf FA, Gillebert TC, Klein AL, Lancellotti P, Marino P, Oh JK, Popescu BA, Waggoner AD. Recommendations for the Evaluation of Left Ventricular Diastolic Function by Echocardiography: An Update from the American Society of Echocardiography and the European Association of Cardiovascular Imaging. J Am Soc Echocardiogr 2016;**29**(4):277-314.

For peer review only

38. Teichholz LE, Cohen MV, Sonnenblick EH, Gorlin R. Study of left ventricular geometry and function by B-scan ultrasonography in patients with and without asynergy. N Engl J Med 1974;**291**(23):1220-6.
39. Yvonne-Tee GB, Rasool AH, Halim AS, Rahman AR. Reproducibility of different laser Doppler fluximetry parameters of postocclusive reactive hyperemia in human forearm skin. J Pharmacol Toxicol Methods 2005;**52**(2):286-92.
40. Tew GA, Klonizakis M, Crank H, Briers JD, Hodges GJ. Comparison of laser speckle contrast imaging with laser Doppler for assessing microvascular function. Microvasc Res 2011;**82**(3):326-32.
41. Papaioannou TG, Karageorgopoulou TD, Sergentanis TN, Protogerou AD, Psaltopoulou T, Sharman JE, Weber T, Blacher J, Daskalopoulou SS, Wassertheurer S, Khir AW, Vlachopoulos C, Stergiopulos N, Stefanadis C, Nichols WW, Tousoulis D. Accuracy of commercial devices and methods for noninvasive estimation of aortic systolic blood pressure a systematic review and meta-analysis of invasive validation studies. J Hypertens 2016;**34**(7):1237-48.
42. Grillo A, Parati G, Rovina M, Moretti F, Salvi L, Gao L, Baldi C, Sorropago G, Faini A, Millasseau SC, Scalise F, Carretta R, Salvi P. Short-Term Repeatability of Noninvasive Aortic Pulse Wave Velocity Assessment: Comparison Between Methods and Devices. Am J Hypertens 2017;**31**(1):80-88.
43. Hametner B, Wassertheurer S, Kropf J, Mayer C, Eber B, Weber T. Oscillometric estimation of aortic pulse wave velocity: comparison with intra-aortic catheter measurements. Blood Press Monit 2013;**18**(3):173-6.
44. Tanaka H, Monahan KD, Seals DR. Age-predicted maximal heart rate revisited. J Am Coll Cardiol 2001;**37**(1):153-6.
45. Evenson KR, Barakat R, Brown WJ, Dargent-Molina P, Haruna M, Mikkelsen EM, Mottola MF, Owe KM, Rousham EK, Yeo S. Guidelines for Physical Activity during Pregnancy: Comparisons From Around the World. Am J Lifestyle Med 2014;**8**(2):102-21.
46. Kuc S, Wortelboer EJ, van Rijn BB, Franx A, Visser GH, Schielen PC. Evaluation of 7 serum biomarkers and uterine artery Doppler ultrasound for first-trimester prediction of preeclampsia: a systematic review. Obstet Gynecol Surv 2011;**66**(4):225-39.
47. Reijnders IF, Mulders A, Koster MPH, Koning AHJ, Frudiger A, Willemsen SP, Jauniaux E, Burton GJ, Steegers-Theunissen RPM, Steegers EAP. New imaging markers for preconceptional and first-trimester utero-placental vascularization. Placenta 2018;**61**:96-102.
48. Rousian M, Koster MPH, Mulders A, Koning AHJ, Steegers-Theunissen RPM, Steegers EAP. Virtual reality imaging techniques in the study of embryonic and early placental health. Placenta 2018;**64 Suppl 1**:S29-S35.
49. Pooh RK, Maeda K, Kurjak A, Sen C, Ebrashy A, Adra A, Dayyabu AL, Wataganara T, de Sa RA, Stanojevic M. 3D/4D sonography - any safety problem. J Perinat Med 2016;**44**(2):125-9.
50. Barnett SB, Maulik D, International Perinatal Doppler S. Guidelines and recommendations for safe use of Doppler ultrasound in perinatal applications. J Matern Fetal Med 2001;**10**(2):75-84.
51. Veerbeek JH, Post Uiterweer ED, Nikkels PG, Koenen SV, van der Zalm M, Koster MP, Burton GJ, van Rijn BB, Franx A. Biopsy techniques to study the human placental bed. Placenta 2015;**36**(8):775-82.
52. Veerbeek JH, Brouwers L, Koster MP, Koenen SV, van Vliet EO, Nikkels PG, Franx A, van Rijn BB. Spiral artery remodeling and maternal cardiovascular risk: the spiral artery remodeling (SPAR) study. J Hypertens 2016;**34**(8):1570-7.
53. Melchiorre K, Sharma R, Khalil A, Thilaganathan B. Maternal Cardiovascular Function in Normal Pregnancy: Evidence of Maladaptation to Chronic Volume Overload. Hypertension 2016;**67**(4):754-62.
54. Gagliardi G, Tiralongo GM, LoPresti D, Pisani I, Farsetti D, Vasapollo B, Novelli GP, Andreoli A, Valensise H. Screening for pre-eclampsia in the first trimester: role of maternal hemodynamics and bioimpedance in non-obese patients. Ultrasound Obstet Gynecol 2017;**50**(5):584-88.
55. Green P, MacLeod Catriona J. SIMR: an R package for power analysis of generalized linear mixed models by simulation. Methods in Ecology and Evolution 2016;**7**(4):493-98.

For peer review only

56. van Oostwaard MF, Langenveld J, Schuit E, Papatsonis DN, Brown MA, Byaruhanga RN, Bhattacharya S, Campbell DM, Chappell LC, Chiaffarino F, Crippa I, Facchinetti F, Ferrazzani S, Ferrazzi E, Figueiro-Filho EA, Gaugler-Senden IP, Haavaldsen C, Lykke JA, Mbah AK, Oliveira VM, Poston L, Redman CW, Salim R, Thilaganathan B, Vergani P, Zhang J, Steegers EA, Mol BW, Ganzevoort W. Recurrence of hypertensive disorders of pregnancy: an individual patient data metaanalysis. Am J Obstet Gynecol 2015;**212**(5):624 e1-17.
57. Voskamp BJ, Kazemier BM, Ravelli AC, Schaaf J, Mol BW, Pajkrt E. Recurrence of small-for-gestational-age pregnancy: analysis of first and subsequent singleton pregnancies in The Netherlands. Am J Obstet Gynecol 2013;**208**(5):374 e1-6.
58. Mostello D, Catlin TK, Roman L, Holcomb WL, Jr., Leet T. Preeclampsia in the parous woman: who is at risk? Am J Obstet Gynecol 2002;**187**(2):425-9.
59. Evers JL. Female subfertility. Lancet 2002;**360**(9327):151-9.
60. van Balen F, Verdurmen JE, Ketting E. Age, the desire to have a child and cumulative pregnancy rate. Hum Reprod 1997;**12**(3):623-7.
61. Ammon Avalos L, Galindo C, Li DK. A systematic review to calculate background miscarriage rates using life table analysis. Birth Defects Res A Clin Mol Teratol 2012;**94**(6):417-23.
62. Foo FL, Collins A, McEniery CM, Bennett PR, Wilkinson IB, Lees CC. Preconception and early pregnancy maternal haemodynamic changes in healthy women in relation to pregnancy viability. Hum Reprod 2017;**32**(5):985-92.
63. Bamfo JE, Kametas NA, Chambers JB, Nicolaides KH. Maternal cardiac function in normotensive and pre-eclamptic intrauterine growth restriction. Ultrasound Obstet Gynecol 2008;**32**(5):682-6.
64. De Paco C, Kametas N, Rencoret G, Strobl I, Nicolaides KH. Maternal cardiac output between 11 and 13 weeks of gestation in the prediction of preeclampsia and small for gestational age. Obstet Gynecol 2008;**111**(2 Pt 1):292-300.
65. Ferrazzi E, Stampalija T, Monasta L, Di Martino D, Vonck S, Gyselaers W. Maternal hemodynamics: a method to classify hypertensive disorders of pregnancy. American Journal of Obstetrics and Gynecology 2018;**218**(1):124.e1-24.e11.
66. Khalil A, Akolekar R, Syngelaki A, Elkhouli M, Nicolaides KH. Maternal hemodynamics in normal pregnancies at 11-13 weeks' gestation. Fetal Diagn Ther 2012;**32**(3):179-85.
67. Mannaerts D, Faes E, Gielis J, Van Craenenbroeck E, Cos P, Spaanderman M, Gyselaers W, Cornette J, Jacquemyn Y. Oxidative stress and endothelial function in normal pregnancy versus pre-eclampsia, a combined longitudinal and case control study. BMC Pregnancy Childbirth 2018;**18**(1):60.
68. Verlohren S, Perschel FH, Thilaganathan B, Droge LA, Henrich W, Busjahn A, Khalil A. Angiogenic Markers and Cardiovascular Indices in the Prediction of Hypertensive Disorders of Pregnancy. Hypertension 2017;**69**(6):1192-97.

For peer review only

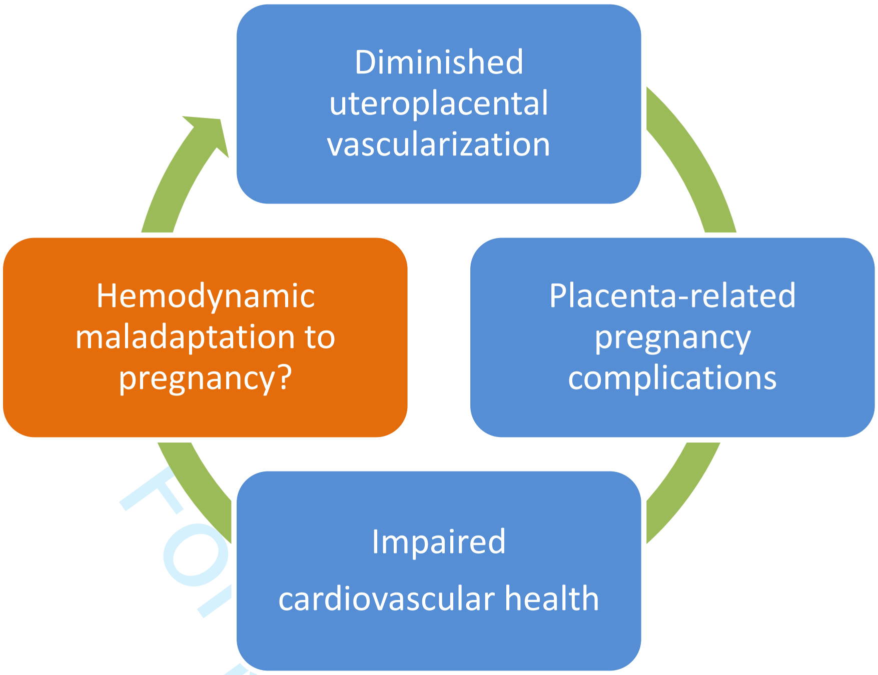

For peer review only

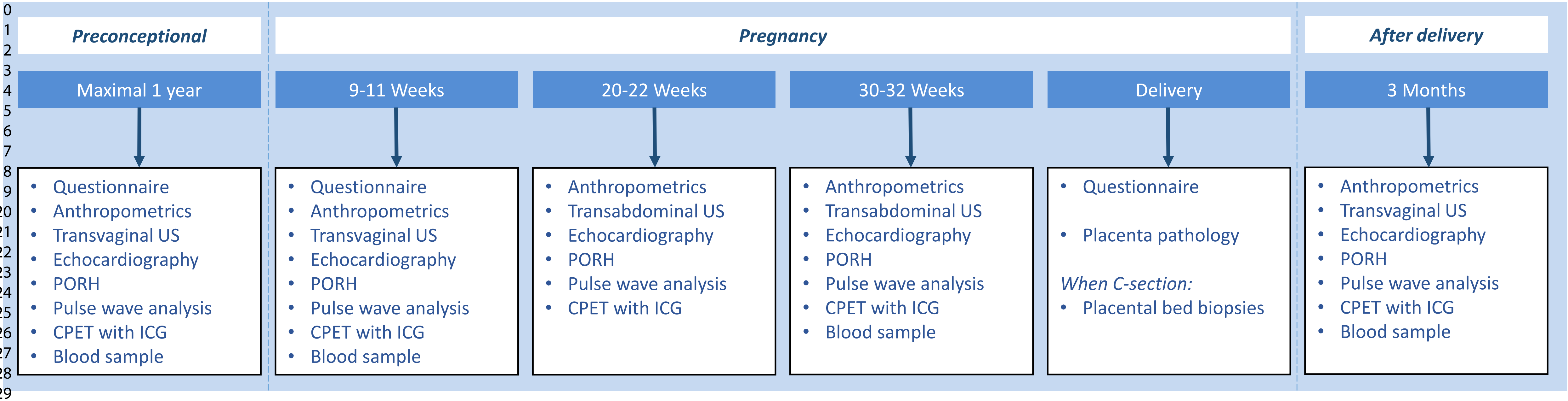
Preconceptional
Pregnancy
After delivery
Maximal 1 year
9-11 Weeks
20-22 Weeks
30-32 Weeks
Delivery
3 Months
Questionnaire
Anthropometrics
Transvaginal US
Echocardiography
PORH
Pulse wave analysis
CPET with ICG
Blood sample
Questionnaire
Anthropometrics
Transvaginal US
Echocardiography
PORH
Pulse wave analysis
CPET with ICG
Blood sample
Anthropometrics
Transabdominal US
Echocardiography
PORH
Pulse wave analysis
CPET with ICG
Anthropometrics
Transabdominal US
Echocardiography
PORH
Pulse wave analysis
CPET with ICG
Blood sample
Questionnaire
Placenta pathology
When C-section:
Placental bed biopsies
Anthropometrics
Transvaginal US
Echocardiography
PORH
Pulse wave analysis
CPET with ICG
Blood sample

BMJ Open

A study protocol for a prospective cohort study to investigate Hemodynamic Adaptation to Pregnancy and Placenta-related Outcome: the HAPPO study

Journal:	*BMJ Open*
Manuscript ID	bmjopen-2019-033083.R1
Article Type:	Protocol
Date Submitted by the Author:	25-Sep-2019
Complete List of Authors:	Bijl, Rianne; Erasmus MC, Obstetrics and Gynaecology<br>Cornette, Jérôme ; Erasmus MC, Obstetrics and Gynaecology<br>van den Bosch, Annemien; Erasmus MC, Cardiology<br>Duvekot, Johannes; Erasmus MC, Obstetrics and Gynaecology<br>Molinger, Jeroen; Erasmus MC, Intensive Care Adults; Duke Medicine, Anesthesiology & Intensive Care medicine, Human Physiology and Pharmacology Lab (HPPL)<br>Willemsen, Sten; Erasmus MC, Obstetrics and Gynaecology; Erasmus MC, Biostatistics<br>Koning, Anton; Erasmus MC, Pathology, Bioinformatics<br>Roos-Hesselink, Jolien; Erasmus MC, Cardiology<br>Franx, Arie; Erasmus MC, Obstetrics and Gynaecology<br>Steegers-Theunissen, Régine; Erasmus MC, Obstetrics and Gynaecology<br>Koster, Maria PH; Erasmus MC, Department of Obstetrics and Gynecology
**Primary Subject Heading**:	Obstetrics and gynaecology
Secondary Subject Heading:	Cardiovascular medicine
Keywords:	Pregnancy complications, Placental development, Preconception, Cardiovascular health, Adaptation

SCHOLARONE™
Manuscripts

A study protocol for a prospective cohort study to investigate Hemodynamic Adaptation to Pregnancy and Placenta-related Outcome: the HAPPO study

Rianne C. Bijl[1], Jérôme M.J. Cornette[1], Annemien E. van den Bosch[2], Johannes J. Duvekot[1], Jeroen Molinger[3,4,5], Sten P. Willemsen[1,6], Anton H.J. Koning[7], Jolien W. Roos-Hesselink[2], Arie Franx[1], Régine P.M. Steegers-Theunissen[1], Maria P.H. Koster[1]

[1] Department of Obstetrics and Gynaecology, Erasmus MC, Rotterdam, the Netherlands
[2] Department of Cardiology, Erasmus MC, Rotterdam, the Netherlands
[3] Department of Intensive Care Adults, Erasmus MC, Rotterdam, the Netherlands
[4] BeLife Clinical Human Performance Centre, Rotterdam, the Netherlands
[5] Department of Anaesthesiology & Intensive Care medicine, Human Physiology and Pharmacology Lab (HPPL). Duke University Medical Centre, Durham, NC, United States
[6] Department of Biostatistics, Erasmus MC, Rotterdam, the Netherlands
[7] Department of Pathology, Bioinformatics, Erasmus MC, Rotterdam, the Netherlands

Correspondence to: dr. M.P.H. Koster, m.p.h.koster@erasmusmc.nl, +31 10 704 0 704
Erasmus MC, University Medical Centre, PO Box 2040, 3000 CA Rotterdam, the Netherlands

Word count: 3535

For peer review only

ABSTRACT

Introduction

The importance of cardiovascular health in relation to pregnancy outcome is increasingly acknowledged. Women who develop certain pregnancy complications, in particular preeclampsia, are at higher risk for future cardiovascular disease. Independent of its outcome, pregnancy requires a substantial adaptive response of the maternal cardiovascular system. In the HAPPO study, we aim to examine longitudinal maternal hemodynamic adaptation to pregnancy from the preconception period onwards. We hypothesize that women who will develop adverse pregnancy outcomes have impaired cardiovascular health before conception, leading to hemodynamic maladaptation to pregnancy and diminished uteroplacental vascular development.

Methods and analysis

In this prospective cohort study embedded in the Rotterdam periconception cohort, 200 women with a history of placenta-related pregnancy complications (high-risk group) and 100 women with an uncomplicated obstetric history (low-risk group) will be included. At five moments (preconception, first-, second- and third trimester, and post-delivery), women will undergo an extensive examination of the macro- and microcirculatory system and uteroplacental vascular development. The main outcome measures are differences in maternal hemodynamic adaptation to pregnancy between women with and without placenta-related pregnancy complications. In a multivariate linear mixed model, the relationship between maternal hemodynamic adaptive parameters, (utero)placental vascularization indices and clinical outcomes (occurrence of pregnancy complications, embryonic and foetal growth trajectories, miscarriage rate, gestational age at delivery, birth weight) will be studied. Subgroup analysis will be performed to study baseline and trajectory differences between high- and low-risk women, independent of subsequent pregnancy outcome.

Ethics and dissemination

This study protocol was approved by the Medical Ethics Committee of the Erasmus MC , Rotterdam, the Netherlands (MEC 2018-150). Results will be disseminated to the medical community by publications in peer-reviewed journals and presentations at scientific

For peer review only

congresses. Also, patient associations will be informed and the public will be informed by dissemination through (social) media.

Registration details

Trial NL7394

Keywords

Pregnancy complications, placental development, preconception, cardiovascular health, adaptation

For peer review only

ARTICLE SUMMARY

Strengths and limitations of this study

- An integrated approach consisting of parallel non-invasive techniques assessing multiple hemodynamic parameters will compile extensive hemodynamic profiles of participants.
- Hemodynamic adaptation to pregnancy will be measured prospectively from the preconception period onwards to three months post-delivery, thereby covering an important window in the reproductive timespan.
- Simultaneous assessment of maternal hemodynamic adaptation to pregnancy and uteroplacental vascular development will provide new insights into the interaction between both mechanisms and the pathophysiology of pregnancy complications.
- Since only multiparous women will be included, conclusions about whether maternal hemodynamic maladaptation is a cause or consequence of previous pregnancy outcome cannot be provided.

For peer review only

INTRODUCTION

Context

Placenta-related pregnancy complications (PPC), such as preeclampsia and/or foetal growth restriction, occur in approximately 10-15% of all pregnancies.[1-3] PPC not only affect perinatal morbidity and mortality but also significantly impact the long-term cardiovascular health of women and their children with associated health care costs for society.[4-6] For example, women with a history of preeclampsia have a two- to sevenfold increased risk to develop ischemic heart disease in later life, depending on the gestational age and severity at onset of preeclampsia.[4] Thus, early identification of women who are at risk for PPC is not only important to enable subsequent personalized prevention and treatment strategies in order to improve reproductive outcome, but also to improve lifelong cardiovascular health. To date, most available screening tests for adverse pregnancy outcome use blood pressure and uterine artery blood flow, which reflect the hemodynamic profile of the mother, and thereby the likelihood of impaired placental development and subsequent adverse events. Nonetheless, these markers are not directly related to cardiac or placental function, which may explain the moderate predictive performances of most prognostic models.[7 8]

Hemodynamic adaptation to pregnancy

Pregnancy requires a physiological adaptive response of the maternal cardiovascular system with a substantial rise in plasma volume and cardiac output (CO).[9] Already in the first trimester of pregnancy, a woman's systemic vascular resistance decreases with 35-40% and her stroke volume increases with 8%.[9] This natural volume overload leads to a reversible, physiological left ventricular dilatation and a possible change in left ventricular diastolic function.[10] In most pregnancies, these changes occur without clinical problems. However, in women who develop PPC, maternal hemodynamic adaptation can be different and diastolic dysfunction is more often present.[11 12]

Moreover, in women with complicated pregnancies, endothelial dysfunction and increased vascular stiffness are also more common.[13 14] The endothelial lining of the vasculature in the human body is of great importance in the regulation of vascular tone. Vascular stiffness increases during normal aging by the replacement of elastin fibres with less elastic collagen. Endothelial function tests and arterial vascular stiffness measurements have already proven to be of importance in predicting cardiovascular disease.[15]

For peer review only

Together, this all supports the hypothesis that in women who develop PPC an increased risk for cardiovascular disease is unmasked by the stressed state of pregnancy.[16 17] However, knowledge on maternal hemodynamic adaptation to pregnancy, as early as the time of conception, is scarce and often only focuses on one or two parameters instead of the complex interaction between macro- and microcirculatory parameters. Only a few studies starting prior to pregnancy have shown hemodynamic changes in, for example, blood pressure, heart rate and CO early in pregnancy.[18-22] An integrated approach using parallel techniques and multiple hemodynamic parameters from the preconception period onwards throughout pregnancy is needed to provide insight in individual (patho)physiological hemodynamic adaptation to pregnancy.

Placental development and function

The adequate establishment of the maternal–foetal vascular interface early in gestation is essential for a healthy pregnancy outcome. Among others, this requires adaptation of the uterine spiral arteries into remodelled vessels to create a low-resistance uteroplacental circulation.[23 24] Failure in the adaptation of the uterine vasculature causes hypoperfusion of the placenta and is widely believed to underlie several pregnancy complications.[25 26] This is substantiated by several changes in histopathology, including decidual vasculopathy, villous hypoplasia and lesions consistent with loss of integrity of the vascular wall of the maternal circulation in delivered placentas of women with PPC.[27-29] However, the aetiology of these placental abnormalities and its relation with maternal cardiovascular health remains unclear.

RESEARCH HYPOTHESIS AND AIM

We hypothesize that impaired maternal cardiovascular health leads to hemodynamic maladaptation to pregnancy, diminished uteroplacental vascular development and subsequent pregnancy complications (Figure 1). The overall aim of the HAPPO study is to examine associations between maternal hemodynamic adaptation to pregnancy and (utero)placental vascular development in women with and without (a history of) pregnancy complications. Therefore, we specified the following objectives:

1. To assess hemodynamic parameters before, during and after pregnancy to gain insight in (patho)physiological maternal hemodynamic adaptation mechanisms;

For peer review only

2. To assess longitudinal utero(placental) vasculature development using state-of-the-art three-dimensional ultrasound and Virtual Reality techniques combined with *in vivo* examination of placental bed biopsies;
3. To construct a multivariate model to study the associations between maternal hemodynamic adaptation to pregnancy, utero(placental) vascular development and PPC.
4. To study differences in baseline hemodynamic parameters and hemodynamic adaptation to pregnancy between women with and without a history of PPC.

STUDY DESIGN

The HAPPO study is a single-centre prospective cohort study, embedded in the ongoing Rotterdam periconception cohort, performed at the department of Obstetrics and Gynaecology of the Erasmus MC, University Medical Centre Rotterdam, the Netherlands.[30]

STUDY POPULATION

We aim to include 200 women with a history of pregnancy complications (i.e. preeclampsia and/or foetal growth restriction [FGR]). Preeclampsia is defined according to the American College of Obstetricians and Gynecologists (ACOG) criteria and FGR is defined conform the Delphi consensus definition described by Gordijn et al.[1 31]

Also, 100 women after uncomplicated pregnancies and (term) deliveries will be included as a low-risk control group. Women are eligible to participate in this study when they have a minimum age of 18 years, the delivery date of their last pregnancy was more than one year ago, they have a current wish to become pregnant and the previous pregnancy was either complicated by PPC *or* the previous pregnancy was uncomplicated. Women who are unable or unwilling to provide informed consent will be excluded from participation. Also, if a woman is currently breastfeeding, she will be excluded from participation since this may influence her vascular condition. The in- and exclusion criteria are shown in Table 1.

For peer review only

Table 1. Inclusion and exclusion criteria for participants in the HAPPO study.

Inclusion criteria	▪ Minimum age of 18 years
	▪ Delivery date of last pregnancy more than 1 year ago
	▪ Previous pregnancy complicated by PPC *or* uncomplicated previous pregnancy
	▪ Current wish to become pregnant
Exclusion criteria	▪ Unable or unwilling to provide informed consent
	▪ Currently breastfeeding

STUDY PROCEDURES/MEASUREMENTS

An overview of all study procedures is provided in Figure 2. The first study visit will be planned when the participating woman has the wish to become pregnant, but before conception takes place. When a pregnancy occurs within one year, a study visit will be planned in each trimester as well as three months post-delivery, which will be the last study visit. All communication with the participants is coordinated by one researcher (RCB) who also performs all study procedures during the study visits.

Questionnaires

Participants of the study will receive a total of three questionnaires: 1) after the preconceptional study visit, participants will receive an online questionnaire on demographic characteristics, medical conditions and lifestyle; 2) preceding the study visit in the first trimester of pregnancy, participants will receive an online questionnaire to address possible changes in medical conditions and lifestyle; 3) after delivery, participants will receive an online follow-up questionnaire on pregnancy and birth outcomes (i.e. gestational age at birth, mode of delivery, birth weight, neonatal sex, pregnancy and/or delivery complications). During the post-delivery visit, the participants will, among others, be asked about current breastfeeding and smoking habits.

Anthropometrics

At each visit, a woman's height, weight, hip and waist circumference will be measured using standardized, validated methods to compose the classical cardiovascular risk factors body mass index (BMI) and waist-to-hip ratio.[32]

For peer review only

Blood sample

Two blood samples (8.5mL serum tube and 10mL EDTA tube) will be obtained at the preconception visit, the first- and third-trimester visit, and the post-delivery visit. All samples will be centrifuged and divided into aliquots of serum, plasma, whole blood and buffy coat and then stored in a -80°C freezer at the trial laboratory at our own facility for future assessment of cardiovascular markers (e.g. CRP and lipids) and markers of placental function (e.g. PlGF and sFlt-1).

Echocardiography

Transthoracic echocardiography has become the preferential method for safe, non-invasive cardiac assessment in pregnant women.[33 34] At all study visits, echocardiography will be performed using an Affiniti 70G ultrasound machine (Philips Healthcare, Andover, United States of America) by a trained researcher (R.C.B.) under the supervision of a cardiologist (A.E.B.). Echocardiograms will be fulfilled according to the recommendations of the European Association of Cardiovascular imaging.[35] CO will be derived from the left ventricle outflow tract velocity time integral (LVOT VTI) and LVOT cross-sectional area (LVOT CSA). From this, systemic vascular resistance will be calculated (i.e. mean blood pressure divided by CO). Left ventricular diastolic function will be assessed by mitral inflow (E/A) and tissue Doppler (E/e') examination. Global left ventricular systolic function will be assessed by using the Teichholz method for ejection fraction calculation.[36]

Post occlusion reactive hyperaemia

One of the first manifestations of an impaired cardiovascular risk profile is endothelial dysfunction of small vessels (microcirculation). Post occlusion reactive hyperaemia (PORH) will be used in this study to assess the endothelial function of the peripheral microvasculature. We use devices from Moor instruments (moorVMS-VASC 2.0, moorVMS-PRES and moorVMS-LDF; Axminster, United Kingdom) to measure microvascular blood flow reactivity following arterial occlusion by using a non-invasive laser Doppler flowmetry (LDF) technique. In resting position, after three minutes of arterial occlusion by a cuff surrounding the upper left arm, the reperfusion of microvascular blood in the skin is measured by the LDF skin probe which is attached to the lower arm. The peak response after the release of the occlusion and the time to recover to baseline reflect endothelial regulated microvascular function.[37 38]

For peer review only

Pulse wave analysis

A cuff-based oscillometric device (Mobil-O-Graph® PWA by I.E.M., Stolberg, Germany) will be used to assess central and peripheral blood pressure, pulse wave velocity and augmentation index (AIx).[39 40] Pulse wave velocity values are derived from an algorithm which integrates age, central systolic blood pressure and data derived from pulse wave analysis into a mathematical model.[41] Higher values of pulse wave velocity indicate higher arterial stiffness and thus a worse vascular condition. The AIx provides additional information on the stiffness of the aorta and is derived from the central pressure waveform.

Cardiopulmonary exercise test with bio-impedance cardiography

To assess the hemodynamic response during physical stress, CO will also be measured using signal morphology impedance cardiography (SM-ICG) using PhysioFlow® PF07 Enduro (Manatec biomedical, Poissy, France). The exercise during which the SM-ICG will be performed, is a cardiopulmonary exercise test (CPET) on a cycle ergometer with a RAMP protocol to 70% of the estimated maximum heart rate according to the Tanaka formula.[42] Moderate exercise limited to 70% of the maximal heart rate is considered safe during pregnancy.[43]

Uteroplacental vascularization measurements

Studies using conventional two-dimensional (2D) Doppler ultrasound have shown that increased vascular resistance of the uterine arteries is associated with an increased risk for subsequent development of PPC.[7 44] Therefore, pulsed wave Doppler measurements of the right and left uterine artery will be performed at all study visits to provide the pulsatility and resistance indices. Moreover, to evaluate the morphology of the uterine and placental vasculature and quantification of perfusion, a 3D power Doppler scan of the uterine (preconception visit) and placental bed (first-trimester visit) vasculature will be performed, also using the Affiniti 70G ultrasound machine. By assessing these 3D datasets in an innovative virtual reality (VR) system (Barco I-Space or 3D desktop), depth perception and three dimensional interaction enables more accurate and detailed quantification of the utero(placental) vascular volumes. At the Erasmus MC there is extensive experience with this

For peer review only

novel technology and the uteroplacental vascular volume measurements have proven to be feasible and reliable.[45 46]

With respect to the risks of performing ultrasound examinations in the first trimester using 3D power Doppler, recommendations as set by the International Society of Ultrasound in Obstetrics and Gynaecology (ISUOG) are followed for the purpose of this study (i.e. thermal index (TI) ≤ 1.0, mechanical index (MI) <1.9 and total exposure time < 10 min). Transvaginal scanning time (preconception and first trimester) and transabdominal scanning time (second and third trimester) will be kept as short as possible according to the ALARA principles.[47 48]

Embryonic and foetal growth parameters

At the first trimester visit, additional parameters of embryonic growth will be measured, i.e. crown-rump length and embryonic volume. The obtained 3D datasets will be visualized in the Barco I-Space or desktop version and offline volume measurements will be performed.

At the second and third trimester visit, additional parameters of foetal growth (head circumference, abdominal circumference, femur length) and pulsed wave Doppler measurements of the umbilical artery and median cerebral artery will be performed by transabdominal ultrasound.

Placental examinations

At delivery, histological samples of *in vivo* placental vasculature will be harvested. The placenta will be collected according to standard procedures and four samples of approximately 3cm*3cm are then fixated in 4% paraformaldehyde for storage. The fixated placenta biopsies will be processed and studied as done in routine placental pathology examination.

In a subgroup of women who undergo a caesarean section (estimated 30% of our study population) at the Erasmus MC, four punch biopsies of the placental bed will be collected, which consist of decidual and myometrial tissue and contain spiral arteries.[49] The samples are directly rinsed with sterile NaCl to wash excessive blood and then fixated in 4% paraformaldehyde for storage and examination. According to our previously published protocol, several markers will be used to score the developmental state of the spiral arteries, identifying typical acute atherosis lesions and evaluating involvement of the immune system.[49 50]

For peer review only

OUTCOME MEASURES

The primary outcome measure of the HAPPO study is the difference in maternal hemodynamic adaptation to pregnancy, expressed as the trajectory of CO assessed by echocardiography before, during and after pregnancy, between women who do and do not develop PPC. CO is one of the most frequently described hemodynamic parameters in pregnancy, alongside with blood pressure. By using transthoracic echocardiography, as a validated and safe method to measure CO, we will obtain consecutive and reliable information on maternal hemodynamic adaptation to pregnancy.[34]

The secondary outcome measures are differences in additional indices of maternal hemodynamic adaptation to pregnancy and utero(placental) vascular development between women who do and do not develop PPC. This includes differences in weight gain during pregnancy, blood pressure, systemic vascular resistance, left ventricular diastolic function, endothelial regulated microvascular function, pulse wave analysis, hemodynamic response to exercise, placental vascular indices, foetal growth parameters (including Doppler measurements) and placenta/placental bed pathology examinations.

SAMPLE SIZE CALCULATION

Since we aim to investigate multiple hemodynamic parameters and literature on this subject is scarce, sample sizes are difficult to calculate. There are a few studies that describe the longitudinal trajectory of CO from preconception until after delivery in uncomplicated pregnancies and a couple of cross-sectional studies that investigated CO in either the first of third trimester of pregnancy.[18-20 51 52] Based on these studies, we expect a difference of approximately 0.5 standard deviation (SD) at each time point and an intraclass correlation coefficient of at least 0.3 between time points in our study. With alpha set at 0.05 and a power of 0.8, simulation for linear mixed model analysis based on these estimates showed that 38 cases (PPC in the anticipated pregnancy) are needed to establish such differences.[53]

Based on literature, we expect a 25% recurrence rate of PPC in the high-risk group and a 2% incidence of PPC in the low-risk group.[54-56] Thus, PPC cases will primarily occur in the high-risk group. For an expected number of 38 cases we would need to follow-up 38/0.25=152 pregnancies in the high-risk group.

For peer review only

Multiparous women who wish to become pregnant who are actively contemplating pregnancy, are assumed to have an approximately 85% chance to become pregnant again within one year.[57 58] The overall miscarriage rate in this study group is estimated to be approximately 10%.[59] With this taken into account, we would need to include 152/(0.85*0.90)=198 high-risk women prior to conception. For comparison with women at low risk for PPC and to increase the number of controls, we will also include 100 low-risk women with an uncomplicated previous pregnancy. With this sample size we expect, based on previous research, to be able to detect differences of approximately 5-10% in other hemodynamic parameters and utero(placental) vasculature development (secondary outcomes).[11 14 18 51 60-66]

STATISTICAL ANALYSIS PLAN

Patient characteristics, medical conditions and lifestyle data will be presented as means [SD] or numbers [percentage] and compared using t-tests and chi-square tests. Hemodynamic parameters will be presented as means [SD] at each time point and compared between groups using t-tests.

Multivariate linear mixed models will be used to analyse the trajectories of maternal hemodynamic parameters over time. We will test whether there is a difference between women who do and do not develop PPC by comparing each trajectory using a likelihood ratio test.

Subgroup analyses will be performed to study (baseline and trajectory) differences between high- and low-risk women, independent of subsequent pregnancy outcome. Bonferroni corrections will be applied to control for multiplicity of secondary outcome parameters.

In case of significant differences in patient characteristics between groups, regression analyses will be adjusted for these characteristics.

DATA COLLECTION, HANDLING AND STORAGE

The study will be performed according to written informed consent procedures which ensures personal data protection and confidentiality. Data will be de-identified (pseudo-anonymized) in order to guarantee the privacy of the participants, the key to the code will be safeguarded by the investigators and will remain on site with the local research team.

For peer review only

All research data will be retained and stored in the HAPPO-database at a secured server at the research institution and it will remain there for at least 15 years after completion of the project. All imaging data will be stored and handled in the same way and according to the same rules and regulations as other patient-related imaging data at Erasmus MC.

Monitoring of data and safety will be performed once a year, according to local protocol for research with negligible risk.

PATIENT AND PUBLIC INVOLVEMENT

Patient and public were not involved in the design of the study. However, at the earliest moment of recruitment, two large patient associations for women who experienced PE and/or HELLP syndrome (i.e. HELLP Stichting [www.hellp.nl] and the Preeclampsia Foundation [www.preeclampsia.org]) were informed and are involved in promoting the HAPPO study.

ETHICS AND DISSEMINATION

The study will be conducted according to the principles of the Declaration of Helsinki (version October 2013) and in accordance with relevant national guidelines, regulations and Acts (e.g. the Medical Research Involving Human Subjects Act [WMO]). The study protocol was approved by the Medical Ethics Committee of the Erasmus MC (MEC-2018-150). The study is registered in the Dutch Trial Registry (NL7394).

The HAPPO study team invites colleagues who are interested in collaboration or data sharing to contact the principal investigators at HAPPO.studie@erasmusmc.nl.

Results of the study will be disseminated to healthcare professionals and to scientific and industrial peers through events, professional organizations and publications in peer-reviewed (preferably open-access) journals as well as presentations at scientific congresses. Also, results will be communicated to women who wish to become pregnant and women who experienced pregnancy complications by informing patient associations and by dissemination via (social) media.

FUTURE IMPLICATIONS

The results of this study will provide more understanding of longitudinal maternal hemodynamic adaptation to pregnancy in relation to utero(placental) vascular development

For peer review only

and its associations with subsequent pregnancy outcome. If we succeed to establish an association between maternal hemodynamic adaptation to pregnancy and subsequent pregnancy outcome, a larger cohort study in nulliparous women will be needed to investigate causal relationships between hemodynamic adaptation to pregnancy and placenta-related outcomes. Hereby, starting points for future possibilities to optimize cardiovascular and placental health by interventions in clinical validation studies will arise. This will enable the development of more accurate, personalized prevention and treatment strategies for women with high-risk pregnancies, from the preconception period onwards. Thereby, we aim to reduce subsequent maternal and perinatal morbidity and mortality in the future, eventually leading to life-long reduced risks and costs of cardiovascular diseases.

DATA AVAILABILITY STATEMENT

There are no data in this work.

AUTHOR'S CONTRIBUTIONS

The rationale and design of the study were conceived by JMJC, JJD, JWRH, RPMST and MPHK. AEB, JM and AHJK contribute their invaluable practical expertise and SPW his excellent statistical expertise. RCB is the executive researcher, supervised by JMJC, AF, and MPHK. MPHK is the principle investigator of the study and responsible for all aspects of the study. RCB and MPHK prepared the first draft of the protocol. All authors have read and approved the final manuscript.

FUNDING STATEMENT

This work is supported by an Erasmus MC Fellowship Grant (2017) and the Peter Joseph Pappas Research Grant from the Preeclampsia Foundation (2019), both awarded to dr. M.P.H. Koster. These sponsors had no involvement in the design of the study.

COMPETING INTEREST STATEMENT

None declared.

For peer review only

FULL REFERENCES

1. ACOG Practice Bulletin No. 202: Gestational Hypertension and Preeclampsia. Obstet Gynecol 2019;**133**(1):e1-e25.
2. ACOG Practice Bulletin No. 204: Fetal Growth Restriction. Obstet Gynecol 2019;**133**(2):e97-e109.
3. Steegers EA, von Dadelszen P, Duvekot JJ, Pijnenborg R. Pre-eclampsia. Lancet 2010;**376**(9741):631-44.
4. Bellamy L, Casas JP, Hingorani AD, Williams DJ. Pre-eclampsia and risk of cardiovascular disease and cancer in later life: systematic review and meta-analysis. Bmj 2007;**335**(7627):974.
5. Veerbeek JH, Hermes W, Breimer AY, van Rijn BB, Koenen SV, Mol BW, Franx A, de Groot CJ, Koster MP. Cardiovascular disease risk factors after early-onset preeclampsia, late-onset preeclampsia, and pregnancy-induced hypertension. Hypertension 2015;**65**(3):600-6.
6. Grandi SM, Filion KB, Yoon S, Ayele HT, Doyle CM, Hutcheon JA, Smith GN, Gore GC, Ray JG, Nerenberg K, Platt RW. Cardiovascular Disease-Related Morbidity and Mortality in Women With a History of Pregnancy Complications. Circulation 2019;**139**(8):1069-79.
7. Townsend R, Khalil A, Premakumar Y, Allotey J, Snell KIE, Chan C, Chappell LC, Hooper R, Green M, Mol BW, Thilaganathan B, Thangaratinam S, Network I. Prediction of pre-eclampsia: review of reviews. Ultrasound Obstet Gynecol 2018.
8. Lamain-de Ruiter M, Kwee A, Naaktgeboren CA, Louhanepessy RD, De Groot I, Evers IM, Groenendaal F, Hering YR, Huisjes AJM, Kirpestein C, Monincx WM, Schielen P, Van 't Zelfde A, Van Oirschot CM, Vankan-Buitelaar SA, Vonk M, Wiegers TA, Zwart JJ, Moons KGM, Franx A, Koster MPH. External validation of prognostic models for preeclampsia in a Dutch multicenter prospective cohort. Hypertens Pregnancy 2019;**38**(2):78-88.
9. Cornette J, Roos-Hesselink JW. Normal Cardiovascular Adaptation to Pregnancy. In: Stergiopoulos K, Brown DL, eds. Evidence-Based Cardiology Consult. London: Springer London, 2014:423-32.
10. Schannwell CM, Zimmermann T, Schneppenheim M, Plehn G, Marx R, Strauer BE. Left ventricular hypertrophy and diastolic dysfunction in healthy pregnant women. Cardiology 2002;**97**(2):73-8.
11. Bosio PM, McKenna PJ, Conroy R, O'Herlihy C. Maternal central hemodynamics in hypertensive disorders of pregnancy. Obstet Gynecol 1999;**94**(6):978-84.
12. Bamfo JE, Kametas NA, Turan O, Khaw A, Nicolaides KH. Maternal cardiac function in fetal growth restriction. Bjog 2006;**113**(7):784-91.
13. Mannaerts D, Faes E, Goovaerts I, Stoop T, Cornette J, Gyselaers W, Spaanderman M, Van Craenenbroeck EM, Jacquemyn Y. Flow-mediated dilation and peripheral arterial tonometry are disturbed in preeclampsia and reflect different aspects of endothelial function. Am J Physiol Regul Integr Comp Physiol 2017;**313**(5):R518-R25.
14. Osman MW, Nath M, Breslin E, Khalil A, Webb DR, Robinson TG, Mousa HA. Association between arterial stiffness and wave reflection with subsequent development of placental-mediated diseases during pregnancy: findings of a systematic review and meta-analysis. J Hypertens 2018;**36**(5):1005-14.
15. Eastabrook G, Aksoy T, Bedell S, Penava D, de Vrijer B. Preeclampsia biomarkers: An assessment of maternal cardiometabolic health. Pregnancy Hypertens 2018;**13**:204-13.
16. Sattar N, Greer IA. Pregnancy complications and maternal cardiovascular risk: opportunities for intervention and screening? Bmj 2002;**325**(7356):157-60.
17. Thilaganathan B, Kalafat E. Cardiovascular System in Preeclampsia and Beyond. Hypertension 2019;**73**(3):522-31.
18. Meah VL, Cockcroft JR, Backx K, Shave R, Stohr EJ. Cardiac output and related haemodynamics during pregnancy: a series of meta-analyses. Heart 2016;**102**(7):518-26.
19. Mahendru AA, Everett TR, Wilkinson IB, Lees CC, McEniery CM. Maternal cardiovascular changes from pre-pregnancy to very early pregnancy. J Hypertens 2012;**30**(11):2168-72.

For peer review only

20. Mahendru AA, Foo FL, McEniery CM, Everett TR, Wilkinson IB, Lees CC. Change in maternal cardiac output from preconception to mid-pregnancy is associated with birth weight in healthy pregnancies. Ultrasound Obstet Gynecol 2017;**49**(1):78-84.
21. Foo FL, Mahendru AA, Masini G, Fraser A, Cacciatore S, MacIntyre DA, McEniery CM, Wilkinson IB, Bennett PR, Lees CC. Association Between Prepregnancy Cardiovascular Function and Subsequent Preeclampsia or Fetal Growth Restriction. Hypertension 2018;**72**(2):442-50.
22. Ghossein-Doha C, Spaanderman ME, Al Doulah R, Van Kuijk SM, Peeters LL. Maternal cardiac adaptation to subsequent pregnancy in formerly pre-eclamptic women according to recurrence of pre-eclampsia. Ultrasound Obstet Gynecol 2016;**47**(1):96-103.
23. Regnault TR, Galan HL, Parker TA, Anthony RV. Placental development in normal and compromised pregnancies-- a review. Placenta 2002;**23 Suppl A**:S119-29.
24. Burton GJ, Jauniaux E. Pathophysiology of placental-derived fetal growth restriction. Am J Obstet Gynecol 2018;**218**(2S):S745-S61.
25. Burton GJ, Charnock-Jones DS, Jauniaux E. Regulation of vascular growth and function in the human placenta. Reproduction 2009;**138**(6):895-902.
26. Fisher SJ. Why is placentation abnormal in preeclampsia? Am J Obstet Gynecol 2015;**213**(4 Suppl):S115-22.
27. Lockwood CJ, Krikun G, Caze R, Rahman M, Buchwalder LF, Schatz F. Decidual cell-expressed tissue factor in human pregnancy and its involvement in hemostasis and preeclampsia-related angiogenesis. Ann N Y Acad Sci 2008;**1127**:67-72.
28. Predoi CG, Grigoriu C, Vladescu R, Mihart AE. Placental damages in preeclampsia - from ultrasound images to histopathological findings. J Med Life 2015;**8 Spec Issue**:62-5.
29. Travaglino A, Raffone A, Saccone G, Migliorini S, Maruotti GM, Esposito G, Mollo A, Martinelli P, Zullo F, D'Armiento M. Placental morphology, apoptosis, angiogenesis and epithelial mechanisms in early-onset preeclampsia. Eur J Obstet Gynecol Reprod Biol 2019;**234**:200-06.
30. Steegers-Theunissen RP, Verheijden-Paulissen JJ, van Uitert EM, Wildhagen MF, Exalto N, Koning AH, Eggink AJ, Duvekot JJ, Laven JS, Tibboel D, Reiss I, Steegers EA. Cohort Profile: The Rotterdam Periconceptional Cohort (Predict Study). Int J Epidemiol 2016;**45**(2):374-81.
31. Gordijn SJ, Beune IM, Thilaganathan B, Papageorghiou A, Baschat AA, Baker PN, Silver RM, Wynia K, Ganzevoort W. Consensus definition of fetal growth restriction: a Delphi procedure. Ultrasound Obstet Gynecol 2016;**48**(3):333-9.
32. de Koning L, Merchant AT, Pogue J, Anand SS. Waist circumference and waist-to-hip ratio as predictors of cardiovascular events: meta-regression analysis of prospective studies. Eur Heart J 2007;**28**(7):850-6.
33. Cornette J, Laker S, Jeffery B, Lombaard H, Alberts A, Rizopoulos D, Roos-Hesselink JW, Pattinson RC. Validation of maternal cardiac output assessed by transthoracic echocardiography against pulmonary artery catheterization in severely ill pregnant women: prospective comparative study and systematic review. Ultrasound Obstet Gynecol 2017;**49**(1):25-31.
34. Bijl RC, Valensise H, Novelli GP, Vasapollo B, Wilkinson I, Thilaganathan B, Stöhr EJ, Lees C, van der Marel CD, Cornette JMJ, on behalf of the International Working Group on Maternal H. Methods and considerations concerning cardiac output measurement in pregnant women: recommendations of the International Working Group on Maternal Hemodynamics. Ultrasound in Obstetrics & Gynecology 2019;**54**(1):35-50.
35. Nagueh SF, Smiseth OA, Appleton CP, Byrd BF, 3rd, Dokainish H, Edvardsen T, Flachskampf FA, Gillebert TC, Klein AL, Lancellotti P, Marino P, Oh JK, Popescu BA, Waggoner AD. Recommendations for the Evaluation of Left Ventricular Diastolic Function by Echocardiography: An Update from the American Society of Echocardiography and the European Association of Cardiovascular Imaging. J Am Soc Echocardiogr 2016;**29**(4):277-314.
36. Teichholz LE, Cohen MV, Sonnenblick EH, Gorlin R. Study of left ventricular geometry and function by B-scan ultrasonography in patients with and without asynergy. N Engl J Med 1974;**291**(23):1220-6.

For peer review only

37. Yvonne-Tee GB, Rasool AH, Halim AS, Rahman AR. Reproducibility of different laser Doppler fluximetry parameters of postocclusive reactive hyperemia in human forearm skin. J Pharmacol Toxicol Methods 2005;**52**(2):286-92.
38. Tew GA, Klonizakis M, Crank H, Briers JD, Hodges GJ. Comparison of laser speckle contrast imaging with laser Doppler for assessing microvascular function. Microvasc Res 2011;**82**(3):326-32.
39. Papaioannou TG, Karageorgopoulou TD, Sergentanis TN, Protogerou AD, Psaltopoulou T, Sharman JE, Weber T, Blacher J, Daskalopoulou SS, Wassertheurer S, Khir AW, Vlachopoulos C, Stergiopulos N, Stefanadis C, Nichols WW, Tousoulis D. Accuracy of commercial devices and methods for noninvasive estimation of aortic systolic blood pressure a systematic review and meta-analysis of invasive validation studies. J Hypertens 2016;**34**(7):1237-48.
40. Grillo A, Parati G, Rovina M, Moretti F, Salvi L, Gao L, Baldi C, Sorropago G, Faini A, Millasseau SC, Scalise F, Carretta R, Salvi P. Short-Term Repeatability of Noninvasive Aortic Pulse Wave Velocity Assessment: Comparison Between Methods and Devices. Am J Hypertens 2017;**31**(1):80-88.
41. Hametner B, Wassertheurer S, Kropf J, Mayer C, Eber B, Weber T. Oscillometric estimation of aortic pulse wave velocity: comparison with intra-aortic catheter measurements. Blood Press Monit 2013;**18**(3):173-6.
42. Tanaka H, Monahan KD, Seals DR. Age-predicted maximal heart rate revisited. J Am Coll Cardiol 2001;**37**(1):153-6.
43. Evenson KR, Barakat R, Brown WJ, Dargent-Molina P, Haruna M, Mikkelsen EM, Mottola MF, Owe KM, Rousham EK, Yeo S. Guidelines for Physical Activity during Pregnancy: Comparisons From Around the World. Am J Lifestyle Med 2014;**8**(2):102-21.
44. Kuc S, Wortelboer EJ, van Rijn BB, Franx A, Visser GH, Schielen PC. Evaluation of 7 serum biomarkers and uterine artery Doppler ultrasound for first-trimester prediction of preeclampsia: a systematic review. Obstet Gynecol Surv 2011;**66**(4):225-39.
45. Reijnders IF, Mulders A, Koster MPH, Koning AHJ, Frudiger A, Willemsen SP, Jauniaux E, Burton GJ, Steegers-Theunissen RPM, Steegers EAP. New imaging markers for preconceptional and first-trimester utero-placental vascularization. Placenta 2018;**61**:96-102.
46. Rousian M, Koster MPH, Mulders A, Koning AHJ, Steegers-Theunissen RPM, Steegers EAP. Virtual reality imaging techniques in the study of embryonic and early placental health. Placenta 2018;**64 Suppl 1**:S29-S35.
47. Pooh RK, Maeda K, Kurjak A, Sen C, Ebrashy A, Adra A, Dayyabu AL, Wataganara T, de Sa RA, Stanojevic M. 3D/4D sonography - any safety problem. J Perinat Med 2016;**44**(2):125-9.
48. Barnett SB, Maulik D, International Perinatal Doppler S. Guidelines and recommendations for safe use of Doppler ultrasound in perinatal applications. J Matern Fetal Med 2001;**10**(2):75-84.
49. Veerbeek JH, Post Uiterweer ED, Nikkels PG, Koenen SV, van der Zalm M, Koster MP, Burton GJ, van Rijn BB, Franx A. Biopsy techniques to study the human placental bed. Placenta 2015;**36**(8):775-82.
50. Veerbeek JH, Brouwers L, Koster MP, Koenen SV, van Vliet EO, Nikkels PG, Franx A, van Rijn BB. Spiral artery remodeling and maternal cardiovascular risk: the spiral artery remodeling (SPAR) study. J Hypertens 2016;**34**(8):1570-7.
51. Melchiorre K, Sharma R, Khalil A, Thilaganathan B. Maternal Cardiovascular Function in Normal Pregnancy: Evidence of Maladaptation to Chronic Volume Overload. Hypertension 2016;**67**(4):754-62.
52. Gagliardi G, Tiralongo GM, LoPresti D, Pisani I, Farsetti D, Vasapollo B, Novelli GP, Andreoli A, Valensise H. Screening for pre-eclampsia in the first trimester: role of maternal hemodynamics and bioimpedance in non-obese patients. Ultrasound Obstet Gynecol 2017;**50**(5):584-88.
53. Green P, MacLeod Catriona J. SIMR: an R package for power analysis of generalized linear mixed models by simulation. Methods in Ecology and Evolution 2016;**7**(4):493-98.
54. van Oostwaard MF, Langenveld J, Schuit E, Papatsonis DN, Brown MA, Byaruhanga RN, Bhattacharya S, Campbell DM, Chappell LC, Chiaffarino F, Crippa I, Facchinetti F, Ferrazzani S, Ferrazzi E, Figueiro-Filho EA, Gaugler-Senden IP, Haavaldsen C, Lykke JA, Mbah AK, Oliveira

For peer review only

VM, Poston L, Redman CW, Salim R, Thilaganathan B, Vergani P, Zhang J, Steegers EA, Mol BW, Ganzevoort W. Recurrence of hypertensive disorders of pregnancy: an individual patient data metaanalysis. Am J Obstet Gynecol 2015;**212**(5):624 e1-17.

55. Voskamp BJ, Kazemier BM, Ravelli AC, Schaaf J, Mol BW, Pajkrt E. Recurrence of small-for-gestational-age pregnancy: analysis of first and subsequent singleton pregnancies in The Netherlands. Am J Obstet Gynecol 2013;**208**(5):374 e1-6.
56. Mostello D, Catlin TK, Roman L, Holcomb WL, Jr., Leet T. Preeclampsia in the parous woman: who is at risk? Am J Obstet Gynecol 2002;**187**(2):425-9.
57. Evers JL. Female subfertility. Lancet 2002;**360**(9327):151-9.
58. van Balen F, Verdurmen JE, Ketting E. Age, the desire to have a child and cumulative pregnancy rate. Hum Reprod 1997;**12**(3):623-7.
59. Ammon Avalos L, Galindo C, Li DK. A systematic review to calculate background miscarriage rates using life table analysis. Birth Defects Res A Clin Mol Teratol 2012;**94**(6):417-23.
60. Foo FL, Collins A, McEniery CM, Bennett PR, Wilkinson IB, Lees CC. Preconception and early pregnancy maternal haemodynamic changes in healthy women in relation to pregnancy viability. Hum Reprod 2017;**32**(5):985-92.
61. Bamfo JE, Kametas NA, Chambers JB, Nicolaides KH. Maternal cardiac function in normotensive and pre-eclamptic intrauterine growth restriction. Ultrasound Obstet Gynecol 2008;**32**(5):682-6.
62. De Paco C, Kametas N, Rencoret G, Strobl I, Nicolaides KH. Maternal cardiac output between 11 and 13 weeks of gestation in the prediction of preeclampsia and small for gestational age. Obstet Gynecol 2008;**111**(2 Pt 1):292-300.
63. Ferrazzi E, Stampalija T, Monasta L, Di Martino D, Vonck S, Gyselaers W. Maternal hemodynamics: a method to classify hypertensive disorders of pregnancy. American Journal of Obstetrics and Gynecology 2018;**218**(1):124.e1-24.e11.
64. Khalil A, Akolekar R, Syngelaki A, Elkhouli M, Nicolaides KH. Maternal hemodynamics in normal pregnancies at 11-13 weeks' gestation. Fetal Diagn Ther 2012;**32**(3):179-85.
65. Mannaerts D, Faes E, Gielis J, Van Craenenbroeck E, Cos P, Spaanderman M, Gyselaers W, Cornette J, Jacquemyn Y. Oxidative stress and endothelial function in normal pregnancy versus pre-eclampsia, a combined longitudinal and case control study. BMC Pregnancy Childbirth 2018;**18**(1):60.
66. Verlohren S, Perschel FH, Thilaganathan B, Droge LA, Henrich W, Busjahn A, Khalil A. Angiogenic Markers and Cardiovascular Indices in the Prediction of Hypertensive Disorders of Pregnancy. Hypertension 2017;**69**(6):1192-97.

For peer review only

<u>Figure 1.</u>

Caption: Schematic hypothesis. Is hemodynamic maladaptation to pregnancy the missing link in the aetiology of placenta-related pregnancy complications?

<u>Figure 2.</u>

Caption: Study procedures. Overview of study visits and examinations to establish cardiovascular function and utero(placental) vascular development from before conception until after delivery.

Legend of figure 2:

US=ultrasound, PORH= post occlusion reactive hyperaemia, CPET=cardiopulmonary exercise test, ICG= impedance cardiography

For peer review only

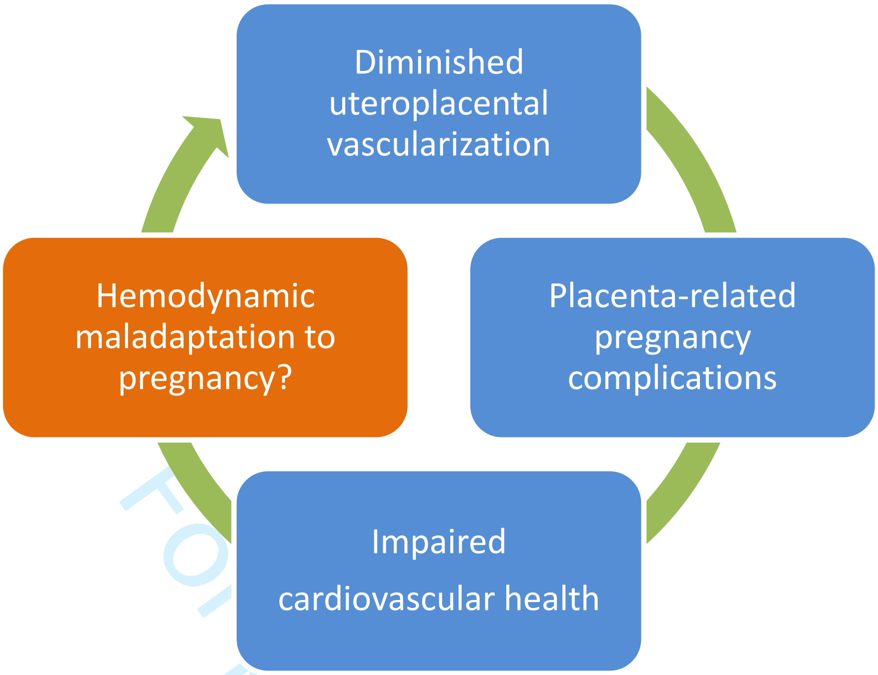

For peer review only

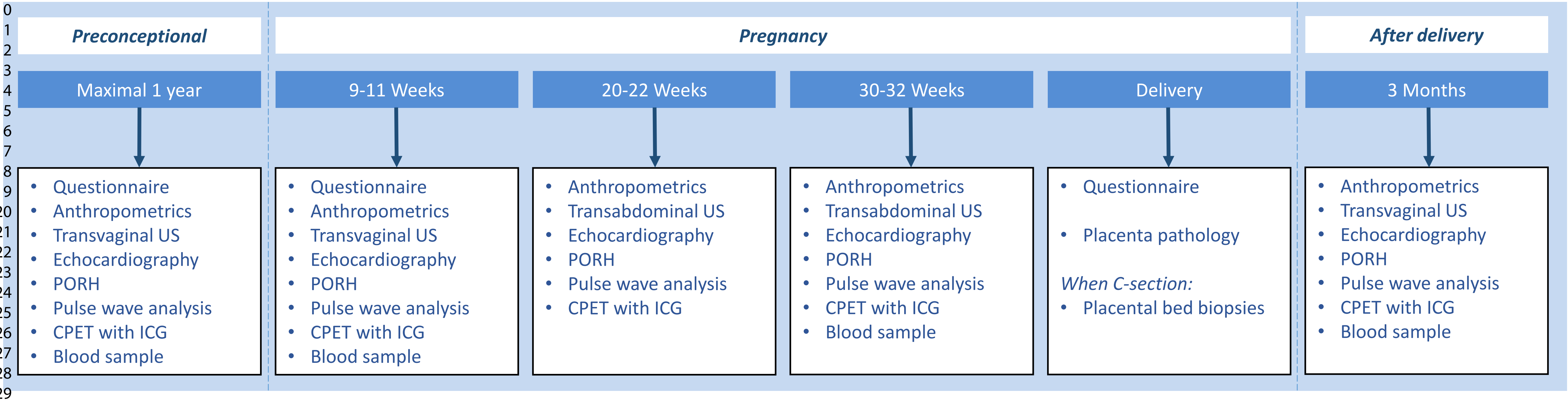
Preconceptional
Pregnancy
After delivery
Maximal 1 year
9-11 Weeks
20-22 Weeks
30-32 Weeks
Delivery
3 Months
• Questionnaire
• Anthropometrics
• Transvaginal US
• Echocardiography
• PORH
• Pulse wave analysis
• CPET with ICG
• Blood sample
• Questionnaire
• Anthropometrics
• Transvaginal US
• Echocardiography
• PORH
• Pulse wave analysis
• CPET with ICG
• Blood sample
• Anthropometrics
• Transabdominal US
• Echocardiography
• PORH
• Pulse wave analysis
• CPET with ICG
• Anthropometrics
• Transabdominal US
• Echocardiography
• PORH
• Pulse wave analysis
• CPET with ICG
• Blood sample
• Questionnaire
• Placenta pathology
When C-section:
• Placental bed biopsies
• Anthropometrics
• Transvaginal US
• Echocardiography
• PORH
• Pulse wave analysis
• CPET with ICG
• Blood sample
